# Association of a Competency-Based Assessment System With Identification of and Support for Medical Residents in Difficulty

**DOI:** 10.1001/jamanetworkopen.2018.4581

**Published:** 2018-11-09

**Authors:** Shelley Ross, Natalia M. Binczyk, Deena M. Hamza, Shirley Schipper, Paul Humphries, Darren Nichols, Michel G. Donoff

**Affiliations:** 1 Department of Family Medicine, University of Alberta, Edmonton, Alberta, Canada

## Abstract

**Importance:**

Competency-based medical education is now established in health professions training. However, critics stress that there is a lack of published outcomes for competency-based medical education or competency-based assessment tools.

**Objective:**

To determine whether competency-based assessment is associated with better identification of and support for residents in difficulty.

**Design, Setting, and Participants:**

This cohort study of secondary data from archived files on 458 family medicine residents (2006-2008 and 2010-2016) was conducted between July 5, 2016, and March 2, 2018, using a large, urban family medicine residency program in Canada.

**Exposures:**

Introduction of the Competency-Based Achievement System (CBAS).

**Main Outcomes and Measures:**

Proportion of residents (1) with at least 1 performance or professionalism flag, (2) receiving flags on multiple distinct rotations, (3) classified as in difficulty, and (4) with flags addressed by the residency program.

**Results:**

Files from 458 residents were reviewed (pre-CBAS: n = 163; 81 [49.7%] women; 90 [55.2%] aged >30 years; 105 [64.4%] Canadian medical graduates; post-CBAS: n = 295; 144 [48.8%] women; 128 [43.4%] aged >30 years; 243 [82.4%] Canadian medical graduates). A significant reduction in the proportion of residents receiving at least 1 flag during training after CBAS implementation was observed (0.38; 95% CI, 0.377-0.383), as well as a significant decrease in the numbers of distinct rotations during which residents received flags on summative assessments (0.24; 95% CI, 0.237-0.243). There was a decrease in the number of residents in difficulty after CBAS (from 0.13 [95% CI, 0.128-0.132] to 0.17 [95% CI, 0.168-0.172]) depending on the strictness of criteria defining a resident in difficulty. Furthermore, there was a significant increase in narrative documentation that a flag was discussed with the resident between the pre-CBAS and post-CBAS conditions (0.18; 95% CI, 0.178-0.183).

**Conclusions and Relevance:**

The CBAS approach to assessment appeared to be associated with better identification of residents in difficulty, facilitating the program’s ability to address learners’ deficiencies in competence. After implementation of CBAS, residents experiencing challenges were better supported and their deficiencies did not recur on later rotations. A key argument for shifting to competency-based medical education is to change assessment approaches; these findings suggest that competency-based assessment may be useful.

## Key Points

**Question:**

Is competency-based assessment associated with changes in rates of identification of and support for residents in difficulty compared with traditional assessment?

**Findings:**

In this cohort study of 458 Canadian medical residents, there were significant reductions in the proportions of residents receiving flagged assessments on multiple rotations, reductions in proportions of residents defined as being in difficulty, and increases in documented evidence identifying that gaps were discussed with the resident following introduction of a competency-based assessment program.

**Meaning:**

Competency-based assessment may contribute to better identification of and support for residents in difficulty.

## Teaser

This cohort study uses secondary data from archived physician resident files from before and after implementation of a competency-based medical education tool to examine the association between this tool and detection and documentation of support for residents’ deficiencies in competence.

## Introduction

Competency-based medical education (CBME) has emerged as a predominant approach to health professions education for the foreseeable future.^1^ Competency-based medical education has been adopted in several countries, including by the Accreditation Council for Graduate Medical Education in the United States,^2^ both the College of Family Physicians of Canada^3^ and the Royal College of Physicians and Surgeons of Canada,^4^ and by accrediting and/or licensing bodies in Scotland,^5^ the Netherlands,^6^ and Australia.^7^

Despite the widespread endorsement of CBME by many accrediting or licensing bodies in health professions training, the shift to CBME is not without controversy.^8,9,10,11,12,13,14^ Although CBME is founded in educational and assessment theory,^15,16^ a prevalent criticism is that there is no evidence that CBME produces safer or more competent physicians than non-CBME approaches. Authors such as Klamen et al,^9^ Boyd et al,^10^ and Whitehead and Kuper^11^ call attention to the gap in the literature of outcomes data for CBME, competency-based assessment tools, or programs of assessment. This pushback against CBME will grow without evidence that CBME frameworks are more effective than traditional medical education in producing competent and safe physicians.^15^

The initial impetus for the CBME movement was a desire to address specific concerns about the varying abilities of graduates of health professions training programs and the potential association of that variation with the quality of care that patients receive.^1,15,16,17,18^ Proponents of CBME argue that competency-based assessment practices can aid with reducing barriers to reporting residents in difficulty through “formative assessments based on identifiable criteria and repeated observations.”^19^ Approaches to assessment in CBME are theorized to improve documentation of feedback shared with residents.^17,20,21^ Increased documentation of more-detailed formative feedback should allow for easier identification of performance patterns, red flags, and trajectory of progress toward competence.^22^ Gruppen at al^23^ recently emphasized the need for studies exploring the association between CBME and the frequency of identification of residents early in training who are not yet ready to be fully trusted in independent practice.

Although most CBME approaches are in the early stages of implementation within training programs, the Competency-Based Achievement System (CBAS),^24^ developed in the Department of Family Medicine at the University of Alberta, Edmonton, Alberta, Canada, has been in place since 2009. Before implementation of CBAS, assessment in the family medicine residency program followed traditional assessment approaches and focused on summative end-of-rotation forms to capture expert judgments of resident competence. Teaching and learning were somewhat disconnected from assessment. In addition, most assessments used forms with rating scales and checklists as the standard tools for capturing observer judgments. Some low-stakes assessment tools (field notes^25^) were used to capture formative feedback shared between residents and observers in the workplace, but their use was not consistent across all preceptors.

The CBAS is designed as programmatic assessment^26,27,28^ predicated on 2 fundamentals: assessment for learning^20,29,30^ and regular formative feedback shared with residents (documented with low-stakes assessment tools).^17,21,25^ The CBAS focuses on direct observation of residents in workplace-based training. In keeping with best practices of workplace-based assessment, CBAS helps to both facilitate and capture experts’ judgment and coaching after observation of learners. The assessment tools in CBAS are designed to allow preceptors to describe what they see the residents do in the workplace and tag or sort their observations according to high-level descriptions of areas of competence in family medicine (professionalism, communication skills, clinical reasoning, medical knowledge, patient-centered care, practice management, procedural skills, and appropriately prioritizing presenting issues).^24^ Although the competencies being assessed were similar for our pre-CBAS vs post-CBAS cohorts, the descriptors of those competencies were changed to enhance clarity and understanding.

The CBAS offers an opportunity to address some of the criticisms of CBME, particularly the need for evidence of proof of concept for CBME (ie, Does CBME result in a different outcome than traditional medical education and assessment?). The CBAS has been the mandated assessment system in a large, 2-year residency program for 8 years, allowing for accumulation of data across cohorts over time. Most of the clinical educators have been consistent for the past 15 years (ie, were teaching and assessing pre- and post-CBAS), which allowed examination of the change in the assessment system rather than a change in those performing the assessment.

In this study, we addressed one of the core assumptions of CBME by examining the extent to which use of competency-based assessment is associated with a change in rates of identification of residents in difficulty compared with traditional assessment. We performed a secondary data analysis of archived resident files from an urban family medicine residency program to compare rates of detection of and documentation of support for residents in difficulty before and after implementation of CBAS.

## Methods

This retrospective, observational cohort study used secondary data analysis of data originally collected as part of the assessment process in the 2-year family medicine residency program at the University of Alberta. Data were extracted from residents’ permanent assessment files and entered into a spreadsheet with random codes replacing names with the express purpose of protecting confidentiality and anonymity of individual residents. This study was approved by the University of Alberta Human Research Ethics Board, and that board also stated that consent was not required for this secondary data analysis study. We adhered to the Strengthening the Reporting of Observational Studies in Epidemiology (STROBE) reporting guideline for reporting observational cohort studies.^31^

Cohorts of residents who started training between 2006 and 2016 were included in the study with the exception of the cohort that began in 2009. Entry cohorts before 2006 were excluded from the study owing to lack of availability of data; the 2009 entry cohort was excluded because this cohort was involved in CBAS pilot implementation. The present study was conducted between July 5, 2016, and March 2, 2018. The University of Alberta has family medicine residents in both urban (approximately 75%) and rural (approximately 25%) streams. Residents who were in the rural training program were excluded because of the heterogeneity of assessment across rural sites during the period of interest.

Demographic data included residents’ sex, residency start year (cohort membership), and age at the time of graduation from the residency program. The medical school from which a resident graduated was used to determine whether the resident was an international medical graduate (resident who completed medical school outside Canada) or a Canadian medical graduate.

Three program directors identified variables (referred to as flags) that indicated that a resident was having difficulty with 1 or more aspects of residency training. These variables are defined in the Box. These flags were identified in the resident’s file and included in the database. For all files with any flag identified, the file was further reviewed for evidence that a program representative had addressed the flag with the resident.

Box. **Definitions of the Variables**

**Flag**

1. Indication of below-average competence on a summative assessment at the end of a rotation or on an overall SPR program, or
2. Any summative assessment that indicated a demonstration of unprofessional behavior.

**Flag Addressed**

1. Comments on the SPR suggesting that the flag was discussed with the resident, and/or
2. One of the following items was marked on the SPR:
  Requires focused attention, or
  Program attention required (on SPRs completed after 2010), or
  Required remediation (on SPRs completed before 2010), and/or
3. Comments on One45 (an online learning management system for summative assessment forms) from a program representative saying that the flag was addressed with the resident; and/or
4. The resident’s file contained a copy of an email or a note about a telephone call indicating that the flag was discussed with the resident; and/or
5. There was a formal assessment plan review to address the flags; and/or
6. There was evidence of remediation related to the flags (eg, a remediation contract).

**Resident in Difficulty**

1. A resident with more than 3 flags regardless of number of rotations flagged;
2. A resident with flags indicated on final assessments from 2 or more rotations;
3. A resident with flags indicated on final assessments from 3 or more rotations.

Abbreviation: SPR, Summative Progress Report.

### Statistical Analysis

Descriptive statistics were calculated. Residents were grouped by cohort start year into either pre-CBAS condition or post-CBAS condition. Pre-CBAS included the cohorts who started residency in 2006, 2007, and 2008; post-CBAS included the cohorts who started in 2010, 2011, 2012, 2013, and 2014.

To compare the frequency of occurrence of flags between the pre-CBAS and post-CBAS conditions, residents were categorized into either yes (≥1 flag) or no (0 flags). Total numbers of flags on assessments were tabulated for each resident. Because residents could receive multiple flags on assessments from a single rotation, the numbers of distinct rotations during which a resident received a flag on at least 1 competency domain on summative rotation assessments were also tabulated.

Because of the categorical nature of the variables being examined, the χ^2^ test was used to examine differences in flags between the pre-CBAS and post-CBAS cohorts. For all tests, a significance level of a 1-tailed *P* < .05 was set and 95% CIs were calculated. The measures tested were (1) total flags received by residents, (2) specific numbers of flags received by individual residents, and (3) numbers of rotations in which residents received flags. The latter analysis was performed because it is possible for a resident to receive multiple flags on 1 rotation assessment, and this analysis was intended to examine whether there were residents who were identified as having deficiencies across multiple rotations (ie, receiving flags across assessments from multiple separate rotations).

The χ^2^ test was also used to compare differences in the frequency of residents in difficulty before and after CBAS. Three definitions of resident in difficulty were used, with each definition applying more strict criteria to reflect variation in the literature around definitions of resident in difficulty (Box).^32,33,34^ The 3 different definitions were used to ensure that the variations seen were reflective of changes in the numbers of residents in difficulty regardless of how stringent the defining criteria were.

The 95% CIs for differences between proportions were calculated using the Pearson χ^2^ formula.^35^ SPSS, version 25.0 (IBM)^36^ was used to perform all analyses.

## Results

Files of 458 family medicine residents were analyzed (100% of all urban program residents in the cohort years included in this study; pre-CBAS: n = 163; 81 [49.7%] women; 90 [55.2%] aged >30 years; 105 [64.4%] Canadian medical graduates; post-CBAS: n = 295; 144 [48.8%] women; 128 [43.4%] aged >30 years; 243 [82.4%] Canadian medical graduates). The basic demographic characteristics of the residents (Table 1) were similar between the pre-CBAS and post-CBAS cohorts with the exception of the proportion of international medical graduates. The pre-CBAS cohort included a higher proportion of international medical graduates (57 [35.0%]) than did the post-CBAS cohorts (52 [17.6%]). Because of this difference in proportions of international medical graduates, all analyses were performed twice, once with the full data set and once with a data set in which the international medical graduates had been removed. This separate analysis was done to determine whether international medical graduates may be disproportionately represented among residents with flags, which would skew the data. In both sets of analyses, all findings were significant (ie, international medical graduates were not skewing the data). A further check was done using logistic regression to determine whether international medical graduate status was associated with receiving a flagged assessment, but the association was not significant. Given this result, we present all findings herein with the full data set.

**Table 1.** Demographic Characteristics of the Residents Included in the File Reviews

Characteristic	Pre-CBAS Cohorts (2006-2008), No. (%)	Post-CBAS Cohorts (2010-2014), No. (%)
Total residents	163^a^	295^b^
Sex		
Women	81 (49.7)	144 (48.8)
Men	81 (49.7)	151 (51.2)
Age, y		
≤30	72 (44.2)	163 (55.3)
>30	90 (55.2)	128 (43.4)
Medical school location		
Canada	105 (64.4)	243 (82.4)
International^c^	57 (35.0)	52 (17.6)

Abbreviations: CBAS, Competency-Based Achievement System.

^a^ One case is missing age, sex, and international vs Canadian medical graduate status information.

^b^ Four cases are missing age information.

^c^ Attended medical school outside Canada.

Differences in the percentage of residents receiving flags on summative assessments are presented in Table 2. Before CBAS implementation, 44.9% (n = 22) to 50.8% (n = 30) of residents in each cohort received at least 1 flag during training; after CBAS implementation, between 16.1% (n = 10) and 27.0% (n = 27) of residents received at least 1 flagged assessment. In addition, there was a significant decrease in the aggregate proportion of residents who received multiple flags on summative assessments after CBAS (0.38; 95% CI, 0.377-0.383), including an observed difference in proportion of residents who were flagged in 5 or more areas. Prior to CBAS implementation, between 16.3% (n = 8) and 27.1% (n = 16) of residents received 5 or more flags, compared with between 0% and 10.7% (n = 6) in the post-CBAS cohorts (n = 458; χ^2^_25_ = 64.5; *P* < .001). A significant decrease after CBAS was also found in the aggregate numbers of distinct rotations during which residents received flags on summative assessments (0.24; 95% CI, 0.237-0.243), most notably for the proportion of residents who received flags on assessments from 4 or more distinct rotations. Before CBAS, this occurred for between 4.1% (n = 2) and 7.3% (n = 4) of residents in each cohort, but in the post-CBAS cohorts, only 1 resident across all post-CBAS cohorts received flagged assessments from 4 or more distinct rotations (n = 458; χ^2^_7_ = 47.04; *P* < .001).

**Table 2.** Percentages of Residents Who Received a Rating of Less Than Satisfactory on a Competency Domain on Any Summative Assessment Before vs After Implementation of CBAS^a^

Characteristic	Pre-CBAS Cohorts (n = 163)			Post-CBAS Cohorts (n = 295)					Aggregate Difference Between Pre- and Post-CBAS, (95% CI)	Effect Size, Φ	*P* value
	2006-2008 (n = 49)	2007-2009 (n = 59)	2008-2010 (n = 55)	2010-2012 (n = 53)	2011-2013 (n = 56)	2012-2014 (n = 61)	2013-2015 (n = 62)	2014-2016 (n = 63)			
Residents receiving summative assessment(s) with at least 1 flagged area, No. (%)^b^	22 (44.9)	30 (50.8)	27 (49.1)	15 (28.3)	16 (28)	16 (26.2)	10 (16.1)	17 (27.0)	0.38 (0.377-0.383)	0.25	*<*.001
Residents receiving summative assessment(s) by number of flagged areas, No. (%)^c^											
1-2 Flagged areas	9 (18.4)	12 (20.3)	11 (20.0)	6 (11.3)	8 (14.3)	4 (6.6)	4 (6.5)	11 (17.5)	0.38 (0.377-0.383)	0.38	*<*.001
3-4 Flagged areas	5 (10.2)	2 (3.4)	3 (5.5)	3 (5.7)	2 (3.6)	8 (13.1)	6 (9.7)	4 (6.3)			
>5 Flagged areas	8 (16.3)	16 (27.1)	13 (23.6)	6 (11.3)	6 (10.7)	4 (6.6)	0	1 (1.6)			
Distinct rotations with summative assessments with at least 1 flagged area, No. (%)^d^											
1 Rotation	8 (16.3)	13 (22.0)	13 (23.6)	7 (13.2)	8 (14.3)	9 (14.8)	4 (6.5)	13 (20.6)	0.24 (0.237-0.243)	0.32	*<*.001
2 Rotations	7 (14.3)	9 (15.2)	4 (7.3)	4 (7.5)	7 (12.5)	5 (8.2)	6 (9.7)	3 (4.8)			
3 Rotations	3 (6.1)	2 (3.4)	5 (9.1)	3 (5.7)	1 (1.8)	1 (1.6)	0	0			
4 Rotations	2 (4.1)	3 (5.1)	1 (1.8)	0	0	0	0	0			
≥5 Rotations	2 (4.1)	3 (5.1)	4 (7.3)	0	0	1 (1.6)	0	0			

Abbreviation: CBAS, Competency-Based Achievement System.

^a^ Percentages reflect the proportion of residents within their cohort year unless otherwise specified.

^b^ Number (percentage) of residents in difficulty: pre-CBAS, 94 (57.7); post-CBAS, 59 (20.0).

^c^ More than 1 flagged area can be indicated on a single rotation assessment. Number (percentage) of residents in difficulty: pre-CBAS, 94 (57.7); post-CBAS, 58 (20.0).

^d^ Number (percentage) of residents in difficulty: pre-CBAS, 79 (48.5); post-CBAS, 72 (24.4).

Table 3 reports changes in the proportions of residents within each cohort who met the criteria for designation as a resident in difficulty, according to the definitions given. Regardless of definition, a significant decrease in the aggregate proportion of residents designated to be in difficulty was found after CBAS, with the magnitude of the change increasing with the strictness of the definition (definition 1: 0.17; 95% CI, 0.168-0.172; definition 2: 0.17; 95% CI, 0.168-0.172; definition 3: 0.13; 95% CI, 0.128-0.132) (Table 3).

**Table 3.** Proportions of Residents Within Each Cohort Who Met Criteria for Designation of Resident in Difficulty Before vs After Implementation of CBAS^a^

Definition	Pre-CBAS Cohorts (N = 163)			Post-CBAS Cohorts (N = 295)					Aggregate Difference Between Pre- and Post-CBAS (95% CI)	Effect Size, Φ	*P* Value
	2006-2008 (n = 49)	2007-2009 (n = 59)	2008-2010 (n = 55)	2010-2012 (n = 53)	2011-2013 (n = 56)	2012-2014 (n = 61)	2013-2015 (n = 62)	2014-2016 (n = 63)			
>3 Flags, regardless of number of rotations flagged, No. (%)^b^	13 (26.5)	19 (32.2)	16 (29.1)	9 (17.0)	8 (14.3)	12 (19.7)	6 (9.7)	1 (1.6)	0.17 (0.168-0.172)	0.19	*<*.001
Flags indicated on final assessments from ≥2 rotations, No. (%)^c^	14 (28.6)	17 (28.8)	14 (25.5)	7 (13.2)	8 (14.3)	7 (11.5)	6 (9.7)	3 (4.8)	0.17 (0.168-0.172)	0.22	<.001
Flags indicated on final assessments from ≥3 rotations, No. (%)^d^	7 (14.3)	8 (13.6)	10 (18.2)	3 (5.7)	1 (1.8)	1 (1.6)	0	0	0.13 (0.128-0.132)	0.25	*<*.001

^a^ Percentages reflect the proportion of residents within their cohort year unless otherwise specified.

^b^ Number (percentage) of residents in difficulty: pre-CBAS, 48 (29.4); post-CBAS, 36 (12.2).

^c^ Number (percentage) of residents in difficulty: pre-CBAS, 45 (27.6); post-CBAS, 31 (10.5).

^d^ Number (percentage) of residents in difficulty: pre-CBAS, 25 (15.3); post-CBAS, 5 (1.7).

We also analyzed changes between pre-CBAS training and post-CBAS training in the frequency of evidence of documentation that a flag on an assessment had been addressed with the resident (Figure). Results indicated that, for residents who had 1 or more flags on assessments, there was a significant increase in documentation that the flag was discussed with the resident between the pre-CBAS and post-CBAS aggregate conditions (0.18; 95% CI, 0.178-0.183). Before CBAS implementation, between 56.7% (n = 17) and 63.6% (n = 14) of files of residents who had received at least 1 flag included documentation that flag(s) had been addressed with the resident. After CBAS implementation, 62.5% (n = 10) to 100% (n = 17) of files included this documentation (n = 155; χ^2^_2_ = 16.83; *P* < .001).

**Figure.**
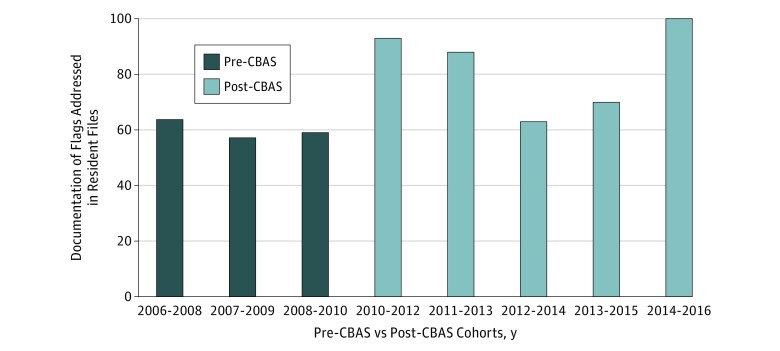
Resident Files That Include Documentation That Flags Were Addressed With the Resident Before and After Implementation of CBAS Proportion by cohort of files for residents with at least 1 flagged assessment where documentation could be found indicating that the flags had been addressed with the resident. CBAS indicates Competency-Based Achievement System.

## Discussion

These findings begin to answer some of the questions raised in the literature about justification for the shift to CBME,^8,9,10,11,12,13,14^ specifically, the need for evidence that CBME is an improvement over traditional medical education. Compared with the traditional assessment approach used in our program before the switch to CBME, competency-based assessment was associated with better identification of residents who encountered difficulties in training and improvement in how concerns about resident competence were addressed.

Since implementation of CBAS, there has been a significant decrease in the proportion of residents receiving at least 1 flag on a summative assessment. Before CBAS, approximately half of the residents in each cohort were flagged at least once during training; after CBAS, fewer than one-third of residents in each cohort were flagged at least once. These findings suggest that ongoing problems continue to be identified in a summative assessment, which is required for any effective assessment system.

There were large decreases in the proportion of residents who were receiving multiple flags. Before CBAS, between 16.3% (n = 8) and 23.6% (n = 13) of residents in each cohort received 5 or more flags on summative assessments. Proportions decreased after CBAS: there were 2 cohorts early in implementation in which approximately 11% of the residents received 5 or more flags (2010-2012 [11.3%] and 2011-2013 [10.7%]), and less than 2% of residents received 5 or more flags in the final 2 cohorts examined (Table 2).

A potential association was found between the decrease in flags on multiple discrete rotations and the reduction in the proportions of residents who met criteria for resident in difficulty. Although the proportion of residents who received a flagged assessment from 1 rotation remained stable across the study period, the proportion of residents who received a flag on assessments from more than 2 rotations decreased to approximately 0% with the exception of 1 resident in the 2012-2014 cohort. In keeping with these findings of a reduction in multiple flagged rotations, there was also a decrease in the number of residents in difficulty regardless of the definition used. Before CBAS, between 14% and 30% of residents in any given cohort could be classified as residents in difficulty (Table 2). After CBAS, a steady decrease in residents in difficulty was seen except in 1 outlier cohort (2012-2014). Even for the outlier cohort, the proportion of residents in difficulty was 6% to 12% lower than in the pre-CBAS cohorts. These findings suggest that the CBAS approach to assessment is associated with better identification of residents who were struggling in 1 or more areas and that those residents were supported so that their deficiencies in competence were not observable on later rotations.

The likelihood that the CBAS approach to assessment is associated with better support of residents who are flagged for deficiencies in competence is further supported by the finding of an increase in documentation showing that flags on summative assessments were discussed with the resident. Identified difficulties should be discussed with learners, but such coaching needs to be facilitated. Before CBAS, 35% to 40% of the residents who received 1 or more flags had no evidence in their files that the flag had been addressed or discussed with them (Figure). After CBAS, 3 of the 5 cohorts examined were found to have documentation that flags on assessments had been discussed with the resident for 88% to 100% of flagged residents. For 2 cohorts (2012-2014 and 2013-2015), the percentage of files with such documentation was lower but higher than the percentage before CBAS.

Overall, this study suggests that a competency-based assessment framework such as CBAS is associated with better identification of residents who have competence gaps. Furthermore, CBAS appears to be associated with better support for residents to address and ameliorate identified gaps. Although the previous assessment approach in this residency program had processes in place that were intended to identify when residents were struggling, the system was ineffective, perhaps because summative assessments were disconnected from daily observations. This failure to identify struggling residents is not unique to this one residency program; rather, this problem has been identified across multiple assessment approaches in medical education and is one of the key justifications for moving to CBME.^1,15,16,17^

It would be possible to dismiss these findings as being a result of improving processes of assessment. However, assessment in a CBME culture must be different,^17,21,37,38,39,40^ and the CBAS approach is fundamentally different from the previous approach to assessment in the residency program examined. In contrast to assessment that focused on capturing end of rotation judgements, the CBAS tools, forms, and processes capture evidence of progress toward competence across clinical experiences, including a representative sampling of the formative feedback shared by the clinical coaches who work with the resident. These low-stakes assessments may reflect and foster learning.

Summative assessments of progress toward competence occur regularly. High stakes in training evaluation reports are completed at the end of every rotation. High-stakes periodic progress reviews occur every 4 months (previously every 6 months). The difference after CBAS is that the periodic progress review is now a shared process in which resident self-reflections on progress toward competence are documented and then discussed between the faculty advisor (competence coach) and the resident, with the low-stakes assessments collected in CBAS used as the evidence base for guided self-assessment.^41^

The transparent nature of assessment in the CBAS framework, as well as the regular provision of formative feedback, has created a culture in which residents in difficulty can be identified early. Two factors contribute to this culture: the proliferation of documented evidence of progress toward competence (which can identify both strengths and gaps) and the regular discussion of the resident’s learning. Addressing a gap, such as a flag, is less stigmatizing in a culture in which supporting residents to be the best physicians that they can be is the focus of assessment. The process of flagging a resident on a summative assessment has not changed: before and after CBAS, a flag means that there are 1 or more topics on which a resident has not demonstrated competence. The difference is that concerns about competence are often discussed with the resident throughout a clinical experience, which means that in many cases, deficiencies are remedied before the final summative assessment at the end of the rotation.

The findings from this study build on the evidence that is emerging that supports the transition to CBME. United States internal medicine residency programs are beginning to publish data about implementation of milestones, which suggests that more information is being collected about the competence of residents in those programs,^42,43^ including increased identification of residents with areas of deficiency.^44^ A competency-based orthopedic residency program at the University of Toronto, Toronto, Ontario, Canada, has been successful in tailoring residency training to ensure that gaps in competencies can be addressed through tailored educational plans and accelerated demonstration of competence can result in reduced time needed to complete training.^45,46^ Evidence is emerging from other pilot programs across North America,^22,47,48,49^ but assessing outcomes takes time.

### Limitations

The study has limitations. Although several hundred resident files across several years were reviewed, the project included residents from only one program and results are reported in aggregate. In addition, although we looked specifically at cohorts before and after implementation of CBAS, other factors may have contributed to the outcomes observed. During the period studied, changes were made in the selection process for the family medicine program, paired with a steady increase in interest in the specialty of family medicine. It is possible that increased competition for family medicine residency positions resulted in admission of higher-achieving candidates. Determining whether our results are a product of CBAS or of higher-caliber residents would be difficult to assess objectively. One area for future research would be to look at rates of residents encountering difficulty in family medicine programs across Canada and comparing those rates with the rates in our program.

There are other future research areas that are relevant to examining the outcome of competency-based assessment, but they are beyond the scope of this study. These include looking at whether the specific competencies that are flagged differ before vs after implementation of CBAS and whether there are clear individual differences within faculty members of what competencies they flag before vs after CBAS.

## Conclusions

The findings from this multiyear comparison of implementation of competency-based assessment and traditional assessment support a proof of concept for CBME. Changing the focus of assessment to an emphasis on direct observation, increased documentation, and assessment for learning may be associated with improved identification of learners who are deficient in 1 or more competency and with how those deficiencies are addressed.
